# Association between serum uric acid levels and colonic diverticulosis in terms of sex

**DOI:** 10.1371/journal.pone.0269978

**Published:** 2022-08-11

**Authors:** Je-Ming Hu, Yu-Tien Chang, Chi-Wei Shih, Chih-Hsiung Hsu, Tzu-Chiao Lin, Chung-Yu Lai, Ming-Hsun Lin, Wei-Liang Chen

**Affiliations:** 1 Division of Colorectal Surgery, Department of Surgery, Tri-Service General Hospital, National Defense Medical Center, Taipei, Taiwan, Republic of China; 2 Graduate Institute of Medical Sciences, National Defense Medical Center, Taipei, Taiwan, Republic of China; 3 Instructor, School of Medicine, National Defense Medical Center, Taipei, Taiwan, Republic of China; 4 School of public health, National Defense Medical Center, Taipei City, Taiwan, Republic of China; 5 Department of General Medicine, Tri-Service General Hospital, and School of Medicine, National Defense Medical Center, Taipei, Taiwan, Republic of China; 6 Department of Internal Medicine, Tri-Service General Hospital, Tri-Service General Hospital, and School of Medicine, National Defense Medical Center, Taipei, Taiwan, Republic of China; 7 Teaching Office, Tri-Service General Hospital, National Defense Medical Center, Taipei, Taiwan, Republic of China; 8 Graduate Institute of Aerospace and Undersea Medicine, National Defense Medical Center, Taipei, Taiwan, Republic of China; 9 Division of Endocrinology and Metabolism, Department of Internal Medicine, Tri-Service General Hospital, National Defense Medical Center, Taipei, Taiwan, Republic of China; 10 Division of Geriatric Medicine, Department of Family and Community Medicine, Tri-Service General, Hospital, and School of Medicine, National Defense Medical Center, Taipei, Taiwan, Republic of China; 11 Division of Family Medicine, Department of Family and Community Medicine, Tri-Service General Hospital, and School of Medicine, National Defense Medical Center, Taipei, Taiwan, Republic of China; Changhua Christian Healthcare System: Changhua Christian Hospital, TAIWAN

## Abstract

**Background:**

The association between elevated serum uric acid (UA) levels and the risk of developing colonic diverticulosis has not yet been investigated. Thus, this cross-sectional study aimed to examine this correlation in individuals from Taiwan.

**Methods:**

From Jan. 1, 2010, to Dec. 31, 2016., approximately 5,605 patients (aged >20 years) from Tri-Service General Hospital who met the inclusion criteria according to colonoscopy and laboratory test findings were included in this research. The correlation between serum UA levels and colonic diverticulosis was investigated via regression analyses.

**Results:**

Participants with elevated serum UA levels were at a higher risk of colonic diverticulosis. The area under the curve for serum UA levels was significantly higher in women than in men (0.651 [95% confidence interval: 0.596–0.707] vs. 0.55 [0.507–0.593]). There were specific trends in female-specific indicators for colonic diverticulosis across increasing quartiles of serum UA levels.

**Conclusions:**

Patients with elevated serum UA levels should be cautious regarding the development of colonic diverticulosis disorder in female. Moreover, prospective studies may provide additional information on the relationship between elevated serum UA levels and colonic diverticulosis.

## 1. Introduction

A recent study showed that the prevalence of colonic diverticulosis is increasing worldwide, particularly in developed western countries [1, 2]. The prevalence is low in some Asian countries; however, it has increased recently [3]. Considering the increasing trend of shifting to a westernized diet in several countries, a previous research showed that lifestyle factors can evidently affect the incidence of the disease [4]. The etiopathogenesis of colonic diverticulosis and diverticular disease is not well understood [5]. The risk factors for colonic diverticulosis include increasing age, [6] male sex, [7] and physical inactivity. Some studies have revealed that excessive alcohol consumption [5] may be a risk factor for the different types of diverticulosis. In addition, metabolic disarrangement can increase the risk of colon diverticular disease [8]. It is important for patients with diverticulosis to manage their disease and other comorbidities. Hence, due to its clinical and economic impact, it is considered one of the most significant conditions in the field of gastroenterology [9]. Therefore, comprehensive evaluation and screening are recommended for populations who are at high risk of colonic diverticulosis.

Epidemiologic studies have shown that the incidence and prevalence of hyperuricemia have increased globally, and more people are at high risk of developing this condition. According to data from the National Health Insurance Research Database (NHIRD) of Taiwan, the prevalence rate (8.2% in men and 2.3% in women) of hyperuricemia is significantly higher in Taiwan than in other countries [10]. An elevated serum uric acid (UA) levels is a major health challenge. Chronic inflammation, a hallmark of hyperuricemia, is significantly associated with comorbidities including hypertension, type II diabetes mellitus (DM), cardiovascular disease (CVD), chronic kidney disease (CKD), urolithiasis, inflammation, dyslipidemia, metabolic syndrome, and even combined Parkinson’s disease and pre-eclampsia [11].

The mechanisms underlying the association between hyperuricemia and colonic diverticulosis remain unknown. We hypothesized that several factors including alcohol abuse, low-fiber diet, [12] and obesity [13, 14] play a role. Hence, hyperuricemia may associate the risk of colonic diverticulosis. However, only few studies have assessed this notion. Thus, this large population-based cohort study aimed to investigate whether there is a correlation between hyperuricemia and the risk of colonic diverticulosis in a population in Taiwan.

## 2. Materials and methods

### 2.1. Ethics statement

The health examination data from the health promotion center of Tri-Service General Hospital (TSGH) comprised comprehensive medical records including laboratory biochemistry data, body composition measurement, and self-reported personal history. The study was conducted according to the protocols of the Institutional Review Board of the Tri-Service General Hospital (TSGH IRB No. 2-107-05-036; Taipei, Taiwan). All procedure were conducted in accordance the guidelines of TSGH. We have obtained patient permission before enrollment by asking them to complete a written informed consent, and approval for the study was granted the IRB of TSGH, Taiwan.

### 2.2. Study design and participants

This retrospective analysis included participants who underwent standard medical screenings including colonoscopy and laboratory testing at the health promotion center of TSGH from Jan. 1, 2010, to Dec. 31, 2016. The study used a self-assessment questionnaire which has been described elsewhere to collect data on lifestyle information. Initially, there were 5,742 participants eligible for the analysis. However, to prevent the effects of underlying comorbidities, we excluded patients receiving urate-lowering medications and with incomplete colonoscopy examination findings, those who could not tolerate colonoscopy or sedation, and those with abnormal findings or missing information on serum UA levels. Finally, there were 5,605 individuals (aged ≥20 years) were enrolled in the final analysis. Serum UA levels were divided according to quartiles to evaluate the relationship between this factor and diverticulosis via a logistic regression analysis. In addition, the dose-dependent effects of serum UA levels on the risk of diverticulosis was investigated. Next, a multivariable logistic regression analysis was performed to examine the association between serum UA levels and diverticulosis in terms of sex after adjusting for other covariates.

### 2.3. Measurement of serum uric acid levels

The participant’s serum UA levels were measured with the Hitachi model 737 multichannel analyzer (Boehringer Mannheim Diagnostics, Indianapolis, IN). Inorganic phosphate determination was performed using ammonium molybdate in an acidic solution to construct ammonium phosphomolybdate, which was quantified according to ultraviolet range (340 nm) using the sample-blanked end point method [15].

### 2.4. Statistical analysis

All statistical analyses were conducted using the Statistical Package for the Social Sciences software version 18.0 (SPSS Inc., Chicago, IL, the USA) for Windows. To assess differences in demographic characteristics, laboratory data, and lifestyle habits, the student’s *t*-test and Pearson’s chi-square test were performed when appropriate. A two-sided *p*-value of ≤0.05 was considered statistically significant. We collected data on demographic characteristics including age, sex, and lifestyle habits (such as smoking history, alcohol consumption, and exercise habits) using self-report questionnaires and identity cards. A receiver operating characteristic (ROC) curve was generated to determine the cutoff point that optimized the sensitivity and specificity of colonic diverticulosis. Serum UA level was divided into quartiles to evaluate the relationship between this factor and diverticulosis via a multiple logistic regression analysis. The participants with the lowest quartiles of serum UA levels were included in the reference group. In addition, the dose-dependent effects of serum UA levels on the risk of diverticulosis were further investigated. Four models were used to adjust covariates, which were as follows: model 1, unadjusted; model 2, age and sex; model 3, model 2 + serum low-density lipoprotein (LDL), creatinine, aspartate aminotransferase (AST), albumin, sodium (Na), and high-sensitivity C-reactive protein (HsCRP) levels; and model 4, model 3 + smoking history, alcohol consumption, and exercise habits. Finally, a multivariate logistic regression analysis was performed to examine the correlation between serum UA levels and colonic diverticulosis in terms of sex after adjusting for other covariates.

## 3. Results

### 3.1. Subsection

#### 3.1.1. Demographic characteristics of the participants

Those with colonic diverticulosis accounted for 6% (n = 328) of the study population. Patients with colonic diverticulosis were significantly older and had higher AST levels. Male participants and smokers were found to have a higher risk of developing colonic diverticulosis (Table 1). However, the other indices were not significant.

**Table 1:** The description statistics of study population.

Item	Colonic diverticulosis		*p* value*
	No (n = 5277)	Yes (n = 328)	
Age (years)	49.8±12.14	57.5±10.34	< 0.001
Serum LDL (mg/dL)	121.15±33.43	124.15±31.93	0.39
Creatinine (mg/dL)	0.82±0.27	0.88±0.27	0.20
AST (U/L)	22.27±12.14	24.99±15.88	< 0.001
Serum Albumin(g/dL)	4.48±0.27	4.46±0.28	0.33
Serum Na(mEq/L)	142.68±2.5	142.66±2.43	0.72
HsCRP(mg/L)	0.24±0.52	0.34±0.73	0.005
Gender (Male)	2496(0.54)	187(0.67)	< 0.001
Smoking	1708(0.33)	132(0.40)	0.004
Alcohol consumption	2259(0.49)	152(0.52)	0.39
Exercise habits	2086(0.39)	129(0.39)	0.94

*Note. serum LDL = low-density lipoprotein; AST = aspartate aminotransferase; serum. Na = serum sodium; HsCRP = high sensitivity C- reactive protein. *p: Chi-square/Fisher exact test on category variables and t-test on continue variables

We analyzed the association between serum UA levels, serum test items, and demographic characteristics and diverticulosis in the four groups according to the quartiles of serum UA levels (cutoff points: 4.6, 5.6, and 6.7 mg/dL). The serum UA level was positively associated with age; serum LDL, creatinine, AST, albumin, and HsCRP levels; sex; smoking history; and alcohol consumption. However, it was negatively correlated with serum Na levels in the Q1–Q3 subgroups based on serum UA levels and was positively associated with exercise habits (Table 2).

**Table 2:** Characteristics of study participants.

	Quartiles of serum urine acid (mg/dL)					
Characteristics of Study Participants	Q1 <4.60 (n = 1473)	Q2 (4.60–5.60) (n = 1439)	Q3 (5.60–6.70) (n = 1337)	Q4 (≧6.70) (n = 1352)	Total (n = 5605)	*P* Value
Continuous variables,mean (SD)						
Age (years)	48.01(11.92)	50.66(12.09)	51.61(12.02)	50.95(12.35)	50.26(12.17)	< 0.001
Height (cm)	160.22(7.268)	163.27(8.766)	166.60(8.817)	168.67(8.142)	164.56(8.866)	< 0.001
Weight (kg)	56.86 (10.424)	63.73 (12.359)	68.93 (11.731)	74.57 (12.471)	65.78 (13.459)	< 0.001
Serum LDL (mg/dL)	113.46(31.27)	120.85(32.47)	123.83(32.78)	127.87(35.25)	121.31(33.35)	< 0.001
Creatinine (mg/dL)	0.69(0.25)	0.79(0.27)	0.88(0.20)	0.97(0.29)	0.83(0.27)	< 0.001
AST (U/L)	20.21(12.60)	21.55(10.62)	22.54(10.70)	25.80(14.66)	22.46(12.41)	< 0.001
Serum Albumin(g/dL)	4.43(0.26)	4.45(0.27)	4.50(0.27)	4.55(0.27)	4.48(0.27)	< 0.001
Serum Na(mEq/L)	142.82(2.64)	142.72(2.55)	142.66(2.45)	142.50(2.31)	142.68(2.50)	0.008
HsCRP(mg/L)	0.20(0.57)	0.24(0.54)	0.26(0.48)	0.30(0.55)	0.25(0.54)	< 0.001
Categorical variables, n (%)						
Gender (Male)	198(7.4)	568(21.2)	887(33.1)	1030(38.4)	2683	< 0.001
Smoking	279(15.2)	411(22.4)	529(28.8)	618(33.6)	1837	< 0.001
Alcohol consumption	479(19.9)	560(23.2)	650(26.9)	724(30.0)	2413	< 0.001
Exercise	556(25.1)	610(27.5)	515(23.2)	535(24.1)	2216	0.059

*Note. serum LDL = low-density lipoprotein; AST = aspartate aminotransferase; serum Na = serum sodium; HsCRP = high sensitivity

C- reactive protein.

*P: Chi-square/ Fisher exact test on category variables and t-test on continue variables.

#### 3.1.2. Dose–response correlation between serum uric acid levels and diverticulosis

**Fig 1:**
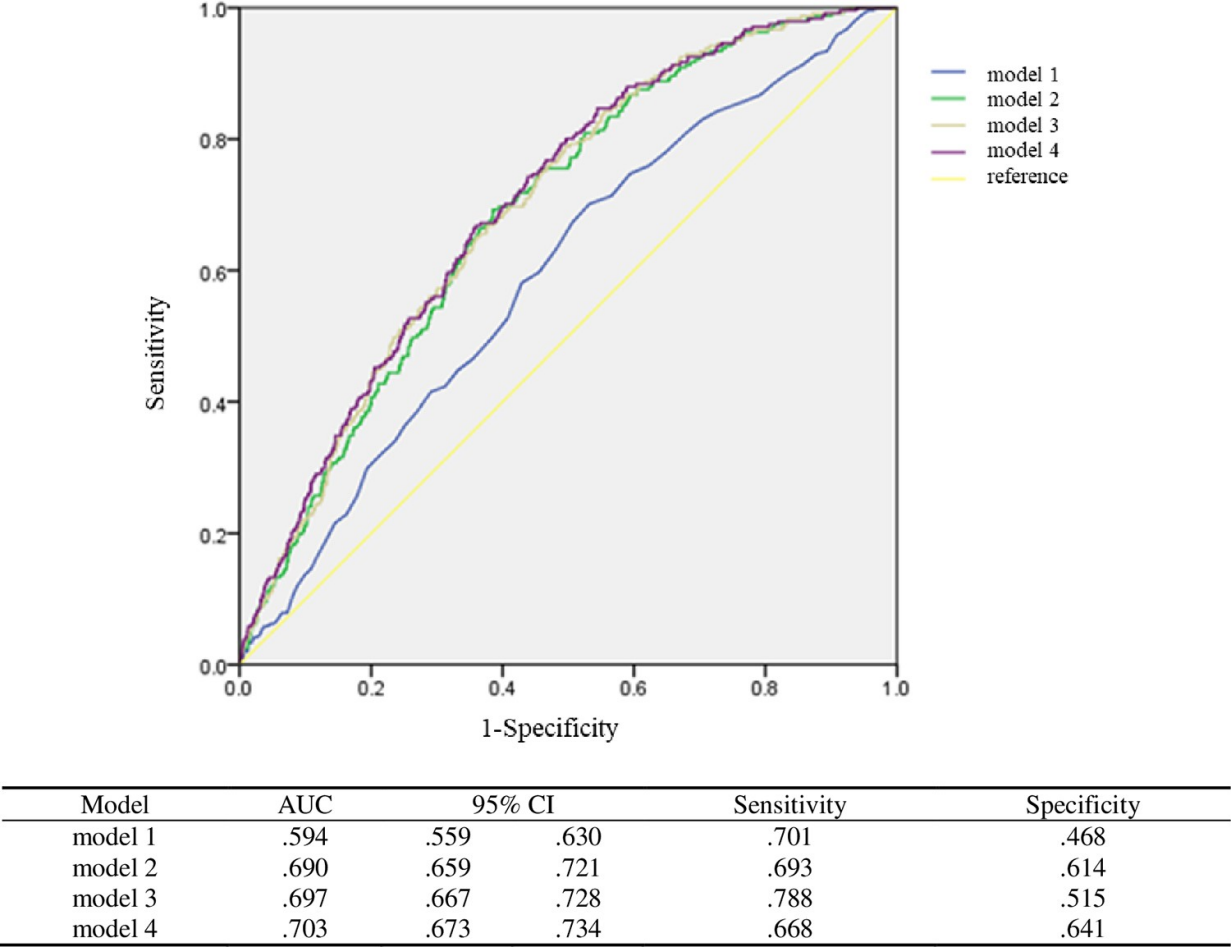
Receiver Operating Characteristic curve (ROC curve) of model 1 to 4. Model 1 = Unadjusted. Model 2 = Model 1 + age, gender. Model 3 = Model 2 + serum LDL, serum creatinine, AST, serum albumin, serum Na, HsCRP. Model 4 = Model 3 + smoking, alcohol consumption, exercise habits.

To further examine the association between continuous variables and the four quartile groups of serum UA levels and diverticulosis, we developed four models via a multivariate logistic regression analysis by adjusting for covariates (Table 3). In all models, a positive association was noted between serum UA levels and the risk of developing colonic diverticulosis, and the odds ratios ranged from 1.153 to 1.217 (*p*<0.05). We classified the serum UA levels according to quantiles, and Q1 was used as the reference group. In model 1, the ORs of the Q3 and Q4 groups were 2.003 (*p* = 0.001) and 2.420 (*p*<0.001), respectively. A dose–response effect was observed. That is, participants with higher UA levels were more likely to develop colonic diverticulosis. We calculated the ROC curves of models 1–4 and found that model 4 had the best predictive power (area under the curve [AUC] = 0.703). There was no statistically significant difference in terms of AUCs in models 2–4. However, they outperformed model 1. Hence, serum UA levels combined with age and sex could have a predictive power for diverticulosis similar to that of models 3 and 4 (Fig 1).

**Table 3:** Association between the serum urine acid and risk of diverticulosis.

	Model a 1		Model a 2		Model a 3		Model a 4	
	Odds ratio (95% CI)	*P* Value	Odds ratio (95% CI)	*P* Value	Odds ratio (95% CI)	*P* Value	Odds ratio (95% CI)	*P* Value
Serum uric acid (as continuous variable)	1.217 (1.121, 1.321)	<0.001	1.166 (1.057, 1.287)	0.002	1.161 (1.048, 1.287)	0.004	1.153 (1.040, 1.278)	0.007
Serum uric acid (as categorial variable)								
Q2 v.s. Q1	1.362 (0.882, 2.103)	0.164	1.136 (0.727, 1.776)	0.575	1.159 (0.739, 1.819)	0.519	1.146 (0.730, 1.798)	0.554
Q3 v.s. Q1	2.003 (1.327, 3.023)	0.001	1.514 (0.968, 2.367)	0.069	1.516 (0.964, 2.382)	0.071	1.470 (0.934, 2.312)	0.096
Q4 v.s. Q1	2.420 (1.617,3.620)	<0.001	1.852 (1.176, 2.916)	0.008	1.844 (1.156, 2.939)	0.010	1.782 (1.117, 2.843)	0.015

*Note. a Adjusted covariates: Model a 1 = Unadjusted, Model a 2 = Model a 1 + age, gender, Model a 3 = Model a 2 + serum LDL, serum creatinine, AST, serum albumin, serum Na, HsCRP, Model a 4 = Model a 3 + smoking, alcohol consumption, exercise habits.

#### 3.1.3. Serum uric acid level as an independent and sex-specific indicator of diverticulosis

**Fig 2:**
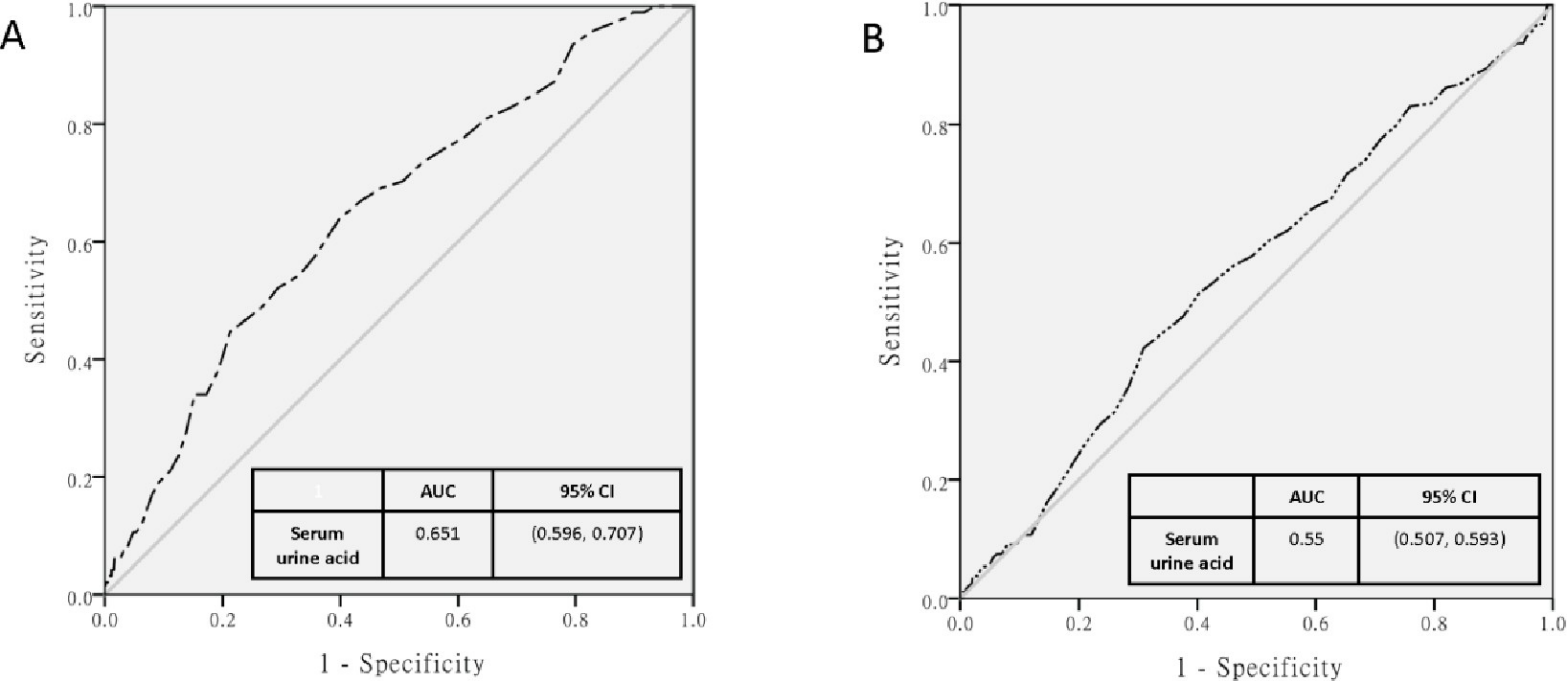
Receiver Operating Characteristic curve (ROC curve) constructed using serum uric acid concentrations (mg/dl) as test variable and diverticulosis as state variable by (A) female and (B) male groups.

We further examined the association between serum UA levels and colonic diverticulosis according to sex. Results showed that serum UA level was positively associated with the risk of developing colonic diverticulosis after adjusting for possible confounders in women. However, there was no association in men. The ORs increased from 1.232 to 1.474 in women (Table 4). The AUC for the serum UA level in women was significantly higher than that in men (0.651 [95% confidence interval, CI: 0.596–0.707] vs 0.55 [0.507–0.593]) (Fig 2).

**Table 4:** Gender difference in association between the serum urine acid and risk of diverticulosis.

	Model ^a^ 1 Odds ratio (95% CI)	*P* Value	Model ^a^ 2 Odds ratio (95% CI)	*P* Value	Model ^a^ 3 Odds ratio (95% CI)	*P* Value	Model ^a^ 4 Odds ratio (95% CI)	*P* Value
Male	1.081 (0.962, 1.215)	0.188	1.112 (0.986, 1.255)	0.083	1.104 (0.975, 1.252)	0.120	1.098 (0.969, 1.245)	0.143
Female	1.474 (1.249, 1.739)	<0.001	1.270 (1.062, 1.519)	<0.001	1.252 (1.035, 1.515)	0.021	1.232 (1.018, 1.492)	0.032

*Note. a Adjusted covariates: Model 1 = Unadjusted; Model 2 = Model 1 + age, gender; Model 3 = Model 2 + serum LDL, serum creatinine, AST, serum albumin, serum Na, HsCRP; Model 4 = Model 3 + smoking, alcohol consumption, exercise habits

#### 3.1.4. Prediction models for diverticulosis using serum uric acid levels

The AUCs of models 1, 2, 3, and 4 were 0.594, 0.690, 0.697, and 0.70, respectively. Models 2–4 have a significantly better prediction efficacy than model 1. However, there was no significant difference in models 2–4. Model 4 had the best AUC after adjusting for smoking history, alcohol consumption, and exercise habits. Sensitivity and specificity were assessed using the Youden’s index. In general, the sensitivity (0.67–0.79)_was higher than the specificity (0.47–0.64) in all models, and model 3 had the highest sensitivity (0.788).

## 4. Discussion

This study showed that women with high UA levels were at high risk of colonic diverticulosis. Therefore, all patients with high serum UA levels should undergo proper evaluations for colonic diverticulosis. To the best of our knowledge, this study first assessed the correlation between serum UA levels and colonic diverticulosis in terms of sex.

Colonic diverticulosis is the most common pathological finding in routine colonoscopy [16]. The incidence of this condition increases substantially with age. That is, it is observed in 15%–30% of individuals aged over 50 years, and two of three people aged over 80 years have this condition [17, 18]. About 10–25% of individuals with colonic diverticulosis present with colonic diverticular disease during their lifetime. In previous studies, the prevalence of asymptomatic colorectal diverticulosis was 13.5% in Taiwan [19]. Our study demonstrated that percentage of diverticulosis was 5.8% (328/5605), which seems to be rather low comparing other studies. A key difference is that our study population was from the health promotion center of TSGH, with relative health group.

People diagnosed with colonic diverticulosis will gradually develop colonic diverticular disease [20]. The exact pathophysiology of colonic diverticulosis and progression to colonic diverticular disease remains unknown. There are several hypotheses showing that diet, motility, microbiome, and inflammation play a role [20]. Most people with colonic diverticulosis do not experience symptoms. When the condition becomes symptomatic, it is referred to as colonic diverticular disease. The symptoms may include constipation, cramps, bloating, and painless bleeding from the rectum. Diverticular disease also includes diverticulitis [21]. The archaic theory of diverticulitis involves high-fat or low-fiber diet and stool lodging in the diverticula, thereby causing trauma, ischemia, and focal perforation [21].

Previous studies have shown that factors such as increasing age [6], male sex [7], and alcohol consumption [5] are correlated with colonic diverticulosis. Intense physical activity decreases the risk of colonic diverticulitis and colonic diverticular hemorrhage [21, 22]. These risk factors were also observed in the current study. In addition, colonic diverticulosis has been associated with low-fiber diet, irregular contraction and muscle spasm in the colon wall, obesity, and intake of specific medications [21]. In addition, several studies have found that hypothyroidism, [23] DM, [23], and arterial hypertension [24] are correlated with a higher risk of colonic diverticulosis disease, but not with colonic diverticulosis in polycystic kidney disease.

Hyperuricemia is an auto-inflammatory disease caused by a purine metabolism disorder, and this results in an abnormally high level of UA in the blood [25]. Data from other studies revealed a correlation between serum UA levels, CVD, CKD, and stroke [26]. However, few studies [7] have assessed the association between serum UA levels and colonic diverticulosis. Several studies have shown that patients with hyperuricemia are at a higher risk of developing colonic diverticular hemorrhage [27]. In approximately 20% of patients, colonic diverticular hemorrhage is associated with hyperuricemia or intake of allopurinol. Therefore, patients with a high UA levels are at high risk of diverticulosis. In a previous study, ROC analysis revealed a low predictive capability with a cutoff point of 5.1 mg/dL (AUC = 71%). However, further sensitivity analysis of possible confounders was not performed. Patients at high risk of diverticulosis should be identified using serum UA levels, and colonoscopy may be recommended to prevent unfavorable outcomes. Therefore, the current study aimed to assess the relationship between serum UA levels and colonic diverticulosis, and a sensitivity analysis was conducted to assess the effects of confounders.

The mechanisms underlying the association between hyperuricemia and colonic diverticulosis remain unknown. We hypothesized that several factors such as alcohol abuse, low-fiber diet, [12], and obesity [13, 14] play a role. In addition, populations with active lifestyle have a lower risk of hyperuricemia than those with sedentary lifestyle [28]. This review focused on recent studies on the bidirectional associations among hyperuricemia, arterial hypertension, progressive renal disease [29, 30], CVD, stroke [31, 32], and DM [33], which are the risk factors of colonic diverticulosis. However, further studies should be conducted to validate the role of lifestyle factors, behavioral problems, and comorbidity on the development of colonic diverticulosis in patients with elevated serum UA levels.

This study showed an association between serum UA levels and colonic diverticulosis after adjusting for factors such as age; sex; serum LDL, creatinine, AST, albumin, Na, and HsCRP levels; smoking history; alcohol consumption; and exercise habits. UA level was an independent predictor of colonic diverticulosis, and the unadjusted model had the highest HR (Table 3). The model including UA level combined with age and sex had a good predictive power for diverticulosis with much less associated factors.

Serum UA level was significantly associated with colonic diverticulosis only in women in each model (Table 4), and this is one of the most important findings of this study. To the best of our knowledge, this research first showed a sex-specific association. A high UA level was found to be associated with a high prevalence of diverticulosis [7]. We calculated the AUCs according to sex (0.651 [95% CI: 0.596–0.707] in women and 0.55 [95% CI: 0.507–0.593] in men). Male participants had higher UA levels than female participants. In male participants, there was a maximum dose–response relationship between serum UA levels and diverticulosis. Hence, there was no correlation between serum UA levels and diverticulosis in men. Several studies have found that microbiota and colonic diverticulosis have a bidirectional association. That is, there was an increased interplay between mucosa-associated microbial communities and the local immune system [34]. The gut microbiome of patient with gout have been dysregulated, with a high number of opportunistic pathogens, and a similar enrichment was also observed in individuals with autoimmune diseases [35]. In addition, one of the possible underlying mechanisms is the imbalance of the microbiota–immune system. Therefore, further studies should be conducted to investigate whether there is a common association between hyperuricemia and colonic diverticulosis.

One possible mechanisms can explain how Taiwanese men did not have relationship between UA and diverticulosis, which women had, including endogenous sex hormones. Colonic diverticulosis was found to be less common in premenopausal women compared to men of the same age in earlier research. After 50 years of age, however, the prevalence of diverticulosis in men and women was the same [36]. This finding implies that ovarian steroid hormones may reduce the risk of diverticulosis in premenopausal women, maybe through beneficial effects on collagen or elastin [37]. Furthermore, previous studies have discovered a significant increase in serum uric acid levels among women aged 50 and older, with both natural and surgical menopause being linked to higher serum UA levels. These data show that menopause and other age-related variables linked to hyperuricaemia are to blame for the rise [38]. UA and colonic diverticulosis share several common links such as endogenous sex hormones. Therefore, further studies are needed to investigate whether there is a common link between UA and colonic diverticulosis in woman.

Although colonic diverticulosis is not a life-threatening disease, it can develop due to severe comorbidities. This study showed that women with hyperuricemia are at high risk of developing colonic diverticulosis, thereby indicating a strong association between the two diseases. Our study had several strengths. First, it used information from the TSGH health management care database, a claims database widely used in academic research. Moreover, it is a universal, single-payer health insurance system comprising comprehensive information, including demographic characteristics of patients, dates of medical visits, and medical services. Second, this database covers a high proportion of people in North Taiwan (8-year data and population-based sample). Hence, selection and participation biases were prevented. Third, the criteria on colonic diverticulosis were defined according to the International Classification of Diseases, Ninth Revision, Clinical Modification, and were monitored and strictly evaluated by the National Health Insurance Administration for Reimbursement Agency.

Although this is a large population-based study, it still has some limitations. First, this research was performed in Taiwan, and it mainly used information on health examination findings. Second, the factors influencing serum UA concentrations such as intake of purine-rich food, UA excretion rate, and UA metabolite levels were not assessed. Third, the use of a cross-sectional design has direct causal inferences, and the longitudinal association between serum UA levels and colonic sensitivity cannot be confirmed because serum UA levels and the associated clinical variables were assessed upon enrollment. In addition, the database used in this study does not have information on the incidence of and comorbidities correlated with colonic diverticulosis and the functional status of patients with high serum UA levels.

## 5. Conclusions

To the best of our knowledge, this study first showed an association between a high risk of colonic diverticulosis and elevated serum UA levels in women. However, further detailed studies should be conducted to validate the correlation between colonic diverticulosis and high serum UA levels. Due to the relatively early onset of colonic diverticulosis and increasing incidence in individuals aged >40 years, early identification of risk factors and symptoms are critical in preventing severe comorbidities correlated to colonic diverticulosis. Moreover, clinicians should follow-up patients with high serum UA levels. However, further prospective studies should be performed to validate our findings. Hence, we recommend meticulous evaluation and aggressive risk reduction for colonic diverticulosis in patients with high serum UA levels. The assessment of serum UA levels was proposed as a new non-invasive method for diagnosing colonic diverticulosis.

## Supporting information

S1 File The physical and mental health questionnaire. (DOCX)
