# Supplementary material for: Association between serum uric acid levels and colonic diverticulosis in terms of sex
Source: PLoS One. 2022 Aug 11;17(8):e0269978. doi: 10.1371/journal.pone.0269978 (PMC9371278; doi:10.1371/journal.pone.0269978)
Supplement: S1 File — (DOCX) [file pone.0269978.s001.docx]

The Physical and Mental Health Questionnaire

| 一、**Basic Information**  1. Name：  2. Gender： □Male □Female  3. ID/Passport Number：  4. Date of Birth(yyyy/mm/dd)：_____ /_____ / _____  5. Date of Employment(yyyy/mm/dd)：_____ / _____ / _____  6. of examination(yyyy/mm/dd)：_____ / _____ / _____ |
| --- |
| 二、**Past Employment Experience**  1. Used to work as __________，Started from (yyyy/mm) _____ / _____，  Ended on (yyyy/mm) _____ / _____，  In total for _____ years _____ months  2. Current work as __________，Started from (yyyy/mm) _____ / _____，  Ended on (yyyy/mm) _____ / _____，  In total for _____ years _____ months  3. In past 1 month, the average weekly working hours: ______ hours  4. In past 6 months, the average weekly working hours: ______ hours |
| 三、**Reason for Examination**  □ New employees  □Regular Examination |
| 四、**Personal Medical History**  Have you ever had underlying chronic diseases：(please mark in front of the appropriate items)  □ Hypertension □ Diabetes Mellitus □ Heart Disease □ Cancer ____  □ Cataract □ Stroke □ Seizure / Epilepsy □ Asthma  □Chronic bronchitis、Emphysema □ Tuberculosis □ Renal Disease  □ Liver Disease □ Anemia □ Otitis Media □ Hearing Impairment  □ Thyroid Disease □ Peptic Ulcer、Gastritis □ Reflux Esophagitis  □ Bone Fracture ____ □ Operation History ____  □ Other Chronic Diseases ____ □ **None of the above** |
| 五、**Life Style Habits**  1. Have you ever been smoking in last 1 month?  □ Never smoke  □ Occasionally used, not everyday  □ Almost every day used，____ cigarettes per day for ____ years.  □ Already quitted for ____ years____ months.  2. Have you ever been using betel nuts in recent 6 months?  □ Never use  □ Occasionally used, not everyday  □ Almost every day used，____ betel nuts per **day** for ____ years.  □ Already quitted for ____ years____ months.  3. Have you ever been drinking in last 1 month?  □ Never drink  □ Occasionally used, not everyday  □ Almost every day used, drink ____ times per **week** with mostly ____ for ____ bottles each time (alcohol brand or name)  □ Already quitted for ____ years____ months.  4. How ofter do you exercise ?  □ I exercise 0-2 times per week  □ I exercise 3-4 times per week  □ I exercise 5 or more times per week  5. On **working days**, your average **daily** sleep hours：____ hours. |
| 六、**Self-awareness Symptoms**  In the previous 3 months, have you frequently suffered from any of the symptoms listed below ? (Please mark in front of the appropriate items)  □ Cough □ Sputum □ Short of breath □ Chest pain □ Palpitations □ Dizziness □ Headache □ Tinnitus □ Fatigue □ Nausea □ Abdominal pain  □ Constipation □ Diarrhea □ Bloody or tarry stool □ Upper backache  □ Lower backache □ Numbness in extremities □ Arthralgia  □ Discomfort while urinating or dysuria □ Frequent urination or polyuria  □ Weakness of extremities □ Body weight loss **>3kg**  □ Other discomfort symptoms □ **None of the above** |
| Notes: 1. Employees are to please fill out basic information, work experience, check period, medical history and subjective symptoms before the health checkup and hand in for health care personnel’s confirmation to effectively screen for illness. If the public institution has provided the basic information and work experience electronic file of the employee to the accredited medical institution, there is no need to ask the employee to fill in it repeatedly. 2. Self-awareness Symptoms : Please check the subjects according to their actual symptoms |
| ========【This part is to be fill out by medical personnel】======== |
| 七、**Check item**  1. Height：______cm  2. Weight：______kg，Waist：_____cm  3. Blood Pressure：_____/_____mmHg  4.Eye sight(Corrected)：Left___Right___；Color vision test：□Normal □Abnormal  5. Hearing test：□Normal □Abnormal  6. Physical examination of each body part :  (1) Head and neck (Conjunctiva, Glandula lymphatia, Thyroid）  (2) Respiratory system  (3) Cardiovascular system (Heart rhythm, Cardiac murmur)  (4) Digestive system (Jaundice, Liver, Abdomen)  (5) Nervous System (Senses)  (6) Musculoskeletal system (Limbs)  (7) Skin  (8) inquiry (Self-awareness Symptoms and sleep status)  7. Chest X ray : ___________  8. Urine Examination : Protein ______ Occult blood ______  9. Blood test : Hgb ______ WBC_______  10. Blood biochemical tests : Blood sugar _____ Alanine aminotransferase (ALT) _____  Creatinine ____ Cholesterol ____ Triglyceride ____ HDL ____ LDL ____  11. Other inspections prescribed by the competent authority of the central government _________ |
| 八、Items in need of further care and attention (multiple selections accepted)  □Results are normal; please continue with regular health checkups.  □Abnormal results are found in one/some test(s), so a follow-up checkup is recommended at the __________ division of a medical institution within ( ).  □Abnormal results are found and you are suggested not to work in _______________ operations.(State the reason(s))  □Abnormal results are found and the following work adjustments are recommended (multiple selections accepted)：  □ Shorten working hours (State the reasons: )  □ Change area of work responsibility (State the reasons: )  □ Change work venue(State the reasons: )  □ Other ________(State the reasons: )  □ Other _________________________________ |
| Notes : 1. Physical examination of each body part : The physical examination Doctor should conduct a detailed examination according to the actual condition of each employee. 2. LDL : No testing required during physical examination. 3. People with congenital abnormalities in color discrimination do not need to be tested during regular physical examination. 4. Those who have undergone screening for oral cancer, colorectal cancer, female cervical cancer and female breast cancer may perform the screening with the consent of the employee, and the results of the screening will not be included in the health check-up record. Accredited medical institutions shall handle inspections and reports in accordance with the screening objects, schedule, data declaration, funds and other stipulations stipulated by the competent authority of the central government - Ministry of Health and Welfare (Taiwan). The screening funds shall be paid by the Health Promotion Administration. |
